# Rose Hips, a Valuable Source of Antioxidants to Improve Gingerbread Characteristics

**DOI:** 10.3390/molecules25235659

**Published:** 2020-12-01

**Authors:** Aliona Ghendov-Mosanu, Elena Cristea, Antoanela Patras, Rodica Sturza, Marius Niculaua

**Affiliations:** 1 Department of Oenology and Chemistry, Food Technology, Technical University of Moldova, 9/9 Studentilor St, MD-2045 Chisinau, Moldova; aliona.mosanu@tpa.utm.md (A.G.-M.); elena.cristea@sa.utm.md (E.C.); rodica.sturza@chim.utm.md (R.S.); 2 Department of Sciences, Faculty of Horticulture, “Ion Ionescu de la Brad” University of Agricultural Sciences and Veterinary Medicine of Iasi, 3 Mihail Sadoveanu Alley, 700490 Iasi, Romania; 3 Research Center for Oenology, Romanian Academy, Iasi Branch, 9 Mihail Sadoveanu Alley, 700490 Iasi, Romania; niculaua@gmail.com

**Keywords:** *Rosa canina* L., antioxidant activity, phenolic compounds, carotenoids, bioactive compounds, natural compounds, food

## Abstract

The present study analyzes the complex of bioactive compounds from rose hips pulp powder (RHP) obtained after separating the seeds from *Rosa canina* L. in order to obtain the oil. The extract prepared from RHP was characterized in terms of the total content of polyphenols, flavonoids, cinnamic acids, flavonols, carotenoids, but also the content of individual polyphenols and carotenoids, antioxidant activity, and CIELab color parameters. The effects of some salts, potentially present in foods, and pH variations were examined to predict possible interactions that could occur when adding rosehip pulp as a food component. The results turned out to be a high content of polyphenols, carotenoids and antioxidant activity. The main phenolic components are procyanidin B1, chlorogenic acid, epicatechin, procyanidin B2, gallic acid, salicylic acid, and catechin. The carotenoid complex includes all-*trans*-β-carotene, all-*trans*-lycopene, zeaxanthin, α-cryptoxanthin, β-cryptoxanthin, rubixanthin, *cis*-β-carotene, *cis*-γ-carotene and *cis*-lycopene. The addition of CaCl_2_ and NaCl to the RHP extract reduced the antioxidant activity and the strong acidic environment (pH to 2.5) decreased the antioxidant activity by 29%. The addition of rose hip powder to gingerbread has improved its general characteristics, and increased its antioxidant activity and microbiological stability, the effects of 4% RHP being the most important.

## 1. Introduction

Publications specialized in the analysis of the food industry report that currently Europe is “the fastest growing market for food colorings, driven by natural and organic products” [1]. Even though synthetic colors still outsell natural ones around the globe, Europe is the largest regional market and thus dictates the trends. The increasing consumer appeal for natural ingredients is expected to raise the demand for natural food dyes in the next few years. Furthermore, extracts obtained from well-known foodstuffs are popular with manufacturers because they are considered ingredients and not additives, and do not require an E number, which scares many consumers [1].

Driven by the consumer demand, companies in the United States of America are also starting to look at natural colorants. Natural food dyes are required in the production of dairy products, carbonated drinks, candies, etc., and manufacturers are searching for bright stable colors of all the colors of the rainbow [2].

Different sources are used nowadays to obtain food colors, and one direction of research is the use of berry extracts. Throughout history, wild berries have been appreciated not only for their color, but also for their health effects. A popular species widely used in Europe is the dog rose. The dog rose or rose hip (*Rosa canina* L.) is a shrub native to Europe and western Asia. It has been used for nutrition and medicinal purposes for centuries [3]. Many dishes are prepared using either fresh or dried hips, among which are syrups, soups in Nordic cuisines, jelly, marmalade, yogurt, bread, alcoholic drinks, and herbal teas [4,5,6,7]. The chemical composition of *Rosa canina* L. extracts has been intensively exploited, given their numerous beneficial effects on the human body [8]. The concentration of ascorbic acid in dog rose is higher than in other species, and coupled with its polyphenols, it stabilizes the content of this vitamin in foodstuffs [9]. Moreover, the data obtained by Lattanzio et al. (2011) suggest that the anti-inflammatory properties of *Rosa canina* L. extracts make this plant a potential adjuvant which can serve as a therapeutic tool for the management of inflammatory-related diseases [10].

Numerous other compounds have been identified, including flavonoids, carotenoids and fatty acids. The class of flavonoids, with over 4500 representatives, has been researched the most thoroughly [11]. Moreover, the existence of several types of fatty acids, including essential ones, has been documented [12]. Several studies describe the antibacterial action of rose hip powders against *Staphylococcus aureus*, *Escherichia coli* and *Klebsiella pneumoniae*. The results obtained attest that Gram-positive bacteria are more sensitive to rose hip powder than Gram-negative bacteria [13]. Additionally, the introduction of dog rose powder in a sausage recipe has reduced the growth rate of microorganisms in intentionally contaminated samples [14]. Dairy products, which contain rose hip powder as a natural ingredient, are more resistant to accidental contamination, which makes them safer for consumption [15,16,17,18,19].

Gingerbread biscuits are a flour confectionery with high energetic and nutritional value. This product is characterized by a pleasant taste and aroma, an attractive appearance, and is often consumed by children and adolescents. Usually gingerbreads are obtained from wheat flour, various nutritional additions (sugars, fats, eggs), spices, flour improvers, flavorings, dyes, and water [20]. The use of powder and extracts from rose hip pulp as natural dyes in gingerbread making facilitates the production of quality foodstuffs with increased biological value.

The purpose of this research was to analyze the extracts prepared from rose hip pulp powder (RHP), of the variety “Visocovitaminnii”, by characterizing its content of polyphenols (total and individual), flavonoids, cinnamic acids, flavonols, carotenoids (total and individual), antioxidant activity and CIELab color parameters. The effects of salts and pH on the antioxidant activity and color parameters of RHP extract were analyzed to predict possible interactions that may occur when using RHP as a food constituent. The influence of RHP addition on antioxidant activity, sensory and physicochemical properties, and the shelf life of gingerbreads was analyzed.

## 2. Results and Discussion

### 2.1. Characterization of Rose Hips

The plant raw material will be characterized thoroughly in this section, as this analysis will deepen the understanding of the phenomena observed after its addition in foodstuffs.

The hips were oval, light-red colored, with a sweet and sour taste. The mass of 100 fresh fruits was 190 ± 25 g, with 49.1 ± 0.2% dry weight, 19.7 ± 0.1 Brix total soluble solids in fruit pulp and total titratable acidity 2.9 ± 0.1 g citric acid/100 g fruit.

The contents of total and individual polyphenols, the carotenoids, and the antioxidant activity of dog rose hips powder were analyzed (Table 1). The results show that the plant material had a high content of polyphenols, i.e., 5484 mg gallic acid equivalents (GAE)/100 g, as determined using Folin–Ciocalteu reagent, and 2968 mg GAE/100 g as determined by measuring the absorbance at 280 nm. The same relatively high difference between the results obtained by the two methods was observed and reported in the case of other plant materials, specifically grape marc [21], chokeberry (*Aronia melanocarpa*) [22], and sea buckthorn (*Hippophae rhamnoides*) [23]. The specific interferences of the Folin–Ciocalteu method, which basically measures the reducing potential in solution, are the main cause of such discrepancies. About 2 g/100 g of the polyphenols found in the dry dog rose hip powder were flavonoids.

Demir et al. [24] have also analyzed five different rose hip (*Rosa* L.) species from Turkey, namely *Rosa canina* L., *Rosa dumalis*, *Rosa gallica*, *Rosa dumalis subsp. Boisieri*, and *Rosa hirtissima*. The authors have determined that the contents of total phenolics in the rose hip fruits were influenced by the species, with the highest level in *Rosa brossieri* (52.94 mg/g) and the lowest in *Rosa canina* L. (31.08 mg/g), while the levels of total flavonoids were almost the same in all samples. The antioxidant and antiradical activities were at high levels in all species, with the lowest Ferric Reducing Antioxidant Power (FRAP) value in *Rosa canina* L. However, the differences in the 2,2′-azino-bis-3-ethylbenzthiazoline-6-sulphonic acid (ABTS) radical scavenging activities were not different among the five species. The concentration of polyphenols in musts from *Rosa canina* L. fruits measured by Czyzowska et al. (2015) was 9007 ± 345 mg GAE/L [9]. Erclisi (2007) also reported that the species influences significantly the content of specific bioactive compounds [25]. The authors also mentioned the variations of maturity stage, climatic conditions, and the use of different analytical methods when explaining these differences [24,25], which were reported for other plant materials [22,23].

The main phenolic compounds found in this study were procyanidin B1, chlorogenic acid (*trans*-5-*O*-caffeoylquinic acid), epicatechin, procyanidin B2, gallic acid, salicylic acid, catechin, etc. Demir et al. [24] found similar amounts of procyanidin B2, gallic, ferulic, and chlorogenic acids, but higher quantities of catechin, and no protocatechuic, vanillic, caffeic, coumaric acids, or epicatechin, in rose hips from Turkey. In total, 45 different phenolic compounds were identified by Cunja et al. [3] in hips of Slovenian *Rosa canina* L. The aforementioned authors used HPLC coupled with MS for their study on the variation of dog rose composition during maturation. They also concluded that the presence and the content of individual polyphenols can drastically change depending on the harvest moment. Catechin was also the main polyphenol identified by Türkben et al. [26], while Demir et al. [24] also identified sinapic acid.

Additionally, the carotenoid content was determined in rose hip powder—64.03 mg/100 g dry weight. Skrypnik et al. (2019) reported that almost the same total carotenoid contents, i.e., 0.61 and 0.64 mg/g, respectively, were determined in the rose hip fruits of the varieties *Rosa canina* L. and *Rosa rugosa* [27]. Andersson et al. (2011) reported the total carotenoid contents in four varieties of rose hip fruits, such as *R. rubiginosa*, *R. dumalis*, *R. dumalis hybrid* and *R. spinosissima*, with values between 1020.81 and 297.11 µg/g dry weight [28]. All-*trans*-lycopene, zeaxanthin, all-*trans*-β-carotene, α-cryptoxanthin, rubixanthin and β-cryptoxanthin were the major compounds identified in a saponified extract. The all-*trans*-lycopene level was rather high [29]. Medveckiene et al. (2020) researched the chemical structure of carotenoids in the pulp of five varieties of rose hip fruits and found that lutein and zeaxanthin range between 12.89 and 20.53%, β-carotene between 45.56 and 70.34%, α-carotene between 6.97 and 13.51%, and lycopene between 9.29 and 24.68% of the total amount of carotenoids [30]. Al-Yafeai et al. (2018) studied the profile of carotenoid compounds in saponified extracts of rose hip fruits at different stages of maturation, and identified the following xanthophylls: violaxanthin, lutein, zeaxanthin and rubixanthin, as well as β-carotene and lycopene [31]. The results of the research attest that the rosehip pulp obtained from the variety “Visocovitaminii” presents numerous carotenoids especially important in the human diet.

### 2.2. Influence of Salts and pH on the Antioxidant Activity and Color of Rose Hip Ethanolic Extract

Sodium chloride, potassium nitrate and calcium chloride are mineral salts that are widely used as additives in food production. In particular, NaCl and CaCl_2_ are important for baking and serve most often as taste enhancers. Calcium chloride is also used as an anti-caking agent in flour. Therefore, a study on the influence of these mineral salts on the antioxidant activity and color of rose hip extract is of interest, as they could affect them.

**Figure 1:**
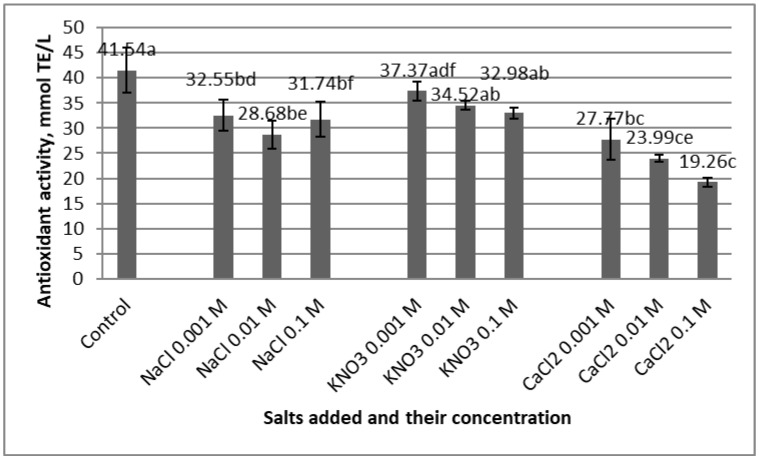
The antioxidant activity of rose hip ethanolic extract in the presence of different salts and different concentrations (standard deviations are based on three replicates; different letters (a–f) designate statistically different results (*p* ≤ 0.05)).

The influences of different salts on the ABTS antioxidant activity and CIELab color parameters of rose hip extracts are presented in Figure 1 and Table 2.

The addition of KNO_3_ produced no statistically significant changes in the value of the antioxidant activity, whereas the other two salts, i.e., CaCl_2_ and NaCl, lowered this parameter. The most drastic decrease was observed in the extract containing CaCl_2_ at 0.1 M concentration. Calcium chloride also decreased the antioxidant activities of grape marc, chokeberry and red cabbage extracts in previous studies [21,22,32].

All the salts produced significant changes on the red/green parameter by degrading the red pigments and shifting the color towards greener tones. The most significant shift was produced by NaCl and CaCl_2_ at all the added concentrations. Furthermore, they lowered the yellowness, shown by the decrease in the b* parameter. The luminosity of the extract was increased by three units on average, while the values for chroma were decreased by three units.

Informational analysis, which shows the dependence between two variables, was also performed. The higher its value, the greater is the dependence. The names of the parameters are given in the nodes of the graph, and the values of the mutual information, measured in bits, are mentioned on the arcs of the graph.

**Figure 2:**
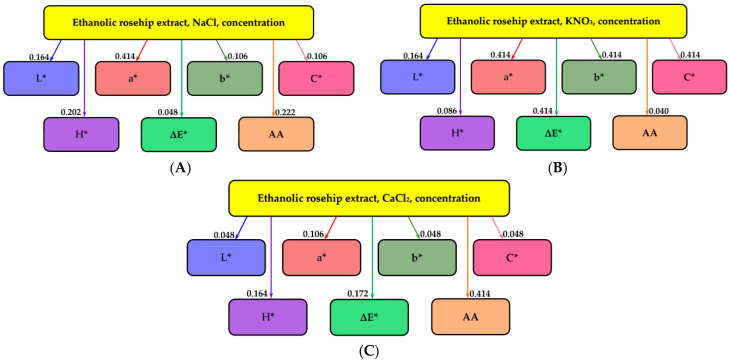
The influences of NaCl (**A**), KNO_3_ (**B**) and CaCl_2_ (**C**) on the CIELab color parameters and the antioxidant activity of rose hip extract. L*—luminosity; a*—red/green component; b*—yellow/blue component; C*—chromaticity; H*—hue angle; ΔE*—overall difference of color; AA—antioxidant activity.

Figure 2A–C present the results of the informational analysis regarding the influence of NaCl, KNO_3_, and CaCl_2_, respectively, on the CIELab color parameters. The quantity of the influencing parameter is the concentration of the respective salt (arranged at the top), while the influenced parameters are the seven mentioned in each graph node.

Figure 2A shows that the concentration of NaCl salt has the most significant influence on the red/green component a* (mutual information 0.414 bits). Following, in descending order of influence, are antioxidant activity AA (0.222 bits), hue angle H* (0.202 bits), luminosity L* (0.164 bits), yellow/blue component b* (0.106 bits), chromaticity C* (0.106 bits), and the overall difference of color ΔE* (0.048 bits).

Figure 2B shows that KNO_3_ influences the most (and equally) the overall color difference ΔE*, yellow/blue component b*, red/green component a*, and chromaticity C* (mutual information 0.414 bits). Following, in descending order of influence, are luminosity L* (0.164 bits), hue angle H* (0.086 bits) and antioxidant activity AA (0.04 bits).

Figure 2C shows that CaCl_2_ mostly influenced antioxidant activity, AA (mutual information 0.414 bits). Following, in descending order of influence, are the overall color difference ΔE* (0.172 bits), hue angle H* (0.164 bits), red/green component a* (0.106 bits), brightness L*, chromaticity C* and yellow/blue component b*, with equal information analysis values of 0.048 bits.

**Figure 3:**
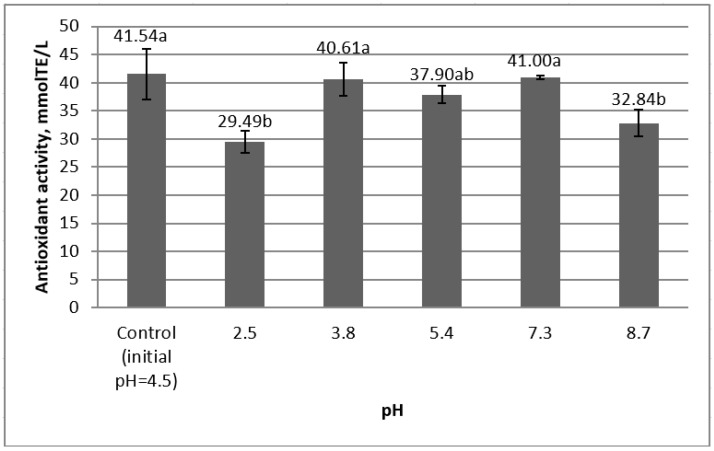
The dependence of antioxidant activity on pH (errors bars represent the standard deviation of three determinations; different letters ^(a,b)^ designate statistically different results (*p* ≤ 0.05)).

Figure 3 presents the values of the antioxidant activity of the rose hip ethanolic extract at different pH values.

Only the most acidic and the most alkaline tested values of pH, namely pH = 2.5 and pH = 8.7, produced a statistically significant effect on the antioxidant activity by decreasing it by around 10 mmol TE/L and 9 mmol TE/L, respectively. However, it must be pointed out that in a non-simultaneous analysis, the difference between the control value and the one for pH = 8.7 only approaches the threshold of significance with *p* = 0.06. The value of this parameter did not suffer any changes at mildly acidic and neutral pH values. It was previously reported that the lowest tested pH = 2.5 decreased significantly the antioxidant activity of chokeberry (*Aronia melanocarpa*) extract [22]. This change in antioxidant activity was observed in the case of grape marc and aronia ethanolic extracts, and was explained by its enhanced ability to donate electrons following the deprotonation and stabilization of polyphenols in alkaline solutions. It is well known that for various hydroxyflavones, the pH-dependent behavior is related to the deprotonation of the hydroxyl fragment, resulting in an increase in antioxidant potential with the appearance of deprotonated forms [33]. Given that the mechanism of the antioxidant activity of radical elimination of the neutral form of hydroxyflavones is generally considered to be the donation of hydrogen ions, this implies not only the ease of radical elimination, but also changes in the mechanism of the antioxidant action in deprotonation. Respectively, a decrease in the antioxidant activity is observed when the pH shifts to acidic values [21,22] (Table 3).

The rose hip extract is relatively stable at different pH values. The most significant effect on L* value was observed at pH = 5.4 and pH = 8.7. At these values, the extracts darkened, and the luminosity shifted from around 96 to around 80 and 93, respectively. Moreover, at pH = 8.7, the redness of the extract increased from −0.1 to 2.4. The same value of pH affected the blue/yellow parameter by increasing the yellowness. As a result, these pH changes, namely the ones at pH 5.4 and 8.7, affected greatly the colorfulness of the extract, which is expressed in the values of ΔE.

**Figure 4:**
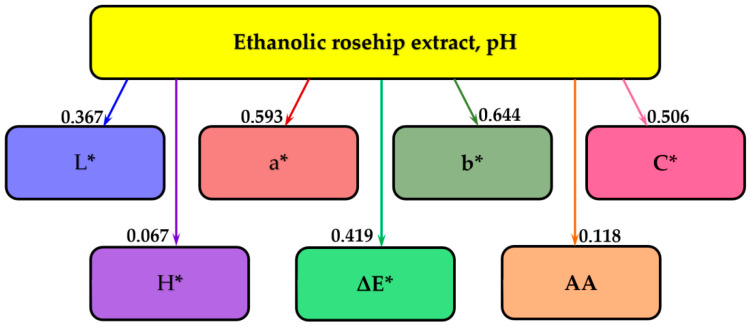
The influence of the pH on the CIELab color parameters and antioxidant parameters of rose hip extract. L*—luminosity; a*—red/green component; b*—yellow/blue component; C*—chromaticity; H*—hue angle; ΔE*—overall difference of color; AA—antioxidant activity.

Figure 4 presents the results of the information analysis, and shows that pH influences most the yellow/blue component b*, with a mutual information of 0.644 bits. This is followed, in descending order, by the red/green component a* (by 0.593 bits), chromaticity C* (0.506 bits), overall color difference ΔE* (0.419 bits), and luminosity L* (0.337 bits). The lowest effects were exhibited on the antioxidant activity AA (0.118 bits) and hue angle H* (0.067 bits).

### 2.3. Gingerbread Characterization

Table 4 presents the physicochemical and microbiological quality parameters, sensory profile, and antioxidant activity of the gingerbreads with the addition of 2% and 4% rose hip powder, and glazed with sugar syrup containing 2% rose hip extract, compared to the control prepared without any addition. The evolution of quality indicators, i.e., mass fraction of dry matter, alkalinity, swelling capacity, and total viable count during storage, was determined on the 1st, 25th and 45th day from the production date.

The obtained results show that a higher concentration of added hip powder contributes to an increase in moisture content compared to the control (10.31%), as follow: 14.01% for 2% RHP and 14.68% for 4% RHP on 1st day. This phenomenon can be explained by the fact that rose hip matter contains pectic substances, which have the ability to bind and retain water in the dough, thus preventing its removal in the baking process. During storage, the moisture of the samples decreased significantly in the first 25 days of storage, specifically in the control sample by 3%, in the 2% RHP sample by 4.3%, and in the 4% RHP sample by 5.5%. In the next 20 days of storage, the moisture content was reduced by 5.5% in the control sample, and in the samples with 2% RHP and 4% RHP by 1.3% and 1.8%, respectively.

It was found that the alkalinity corresponds to the legally acceptable norms and does not exceed the limit of 2 degrees, both in the control and the samples with added rose hip powder [34]. The alkalinity was lower in the samples with rose hip addition compared to the control, most probably due to the organic acids present in the plant matter, which were partially neutralized by the ammonium bicarbonate. As for the variation in the alkalinity during the storage of biscuits, the data show that it decreased insignificantly.

An important feature of flour confectionery is the sorption capacity, which is most often expressed by its swelling capacity. The obtained results showed that the increase in the concentration of rose hip in the fortified gingerbread biscuits raised the values of the swelling capacity compared to the control. During storage for 45 days, this parameter increased 1.03 times in the control, and 1.1 times in the 2% and 4% RHP samples. This is due to the loss of moisture during storage and to the higher content of dietary fiber in the samples with rose hip addition [35].

Previous research has shown that samples with the addition of rose hip powder presented a higher microbiological stability compared to the control. The microbiological load, i.e., total viable count (TVC), was determined to assess microbiological stability. The method includes the quantification of mesophilic aerobic organotrophic bacteria [36]. The reduction of TVC is more important in the case of the addition of 4% hip powder. This can be explained by the antimicrobial properties of phenolic compounds, especially flavonoids and tannins, which prevent the development of microorganisms and allow the stabilization of food [13], possibly by reaction with sulfhydryl groups or by interactions with proteins, with the formation of reactive quinones that can react with amino acids and proteins, inhibiting the synthesis of nucleic acids of both Gram-negative and Gram-positive bacteria [37]. A reverse relationship was observed between total viable count and antioxidant activity; TVC increased during 45 days of storage, although it was kept within tolerable limits, while the antioxidant activity was reduced. It is known that flavonoids, due to the presence of -OH groups, tend to be incorporated into the membrane and cell wall of the microorganism, which leads to changes in their fluidity and permeability and causes a weakening of the membrane potential, which consequently contributes to microbial cell death [38].The presence of biologically active compounds essentially influences the stability and antioxidant capacity of confectionery [39]. It has been found that the inclusion of plant sources rich in antioxidants influences not only microbiological stability, but also antioxidant capacity. The presence of natural antioxidants also decreases the degree of lipid oxidation of products [40]. The DPPH antioxidant activity of gingerbreads, measured in vitro, in the conditions of gastric digestion was investigated. During the 45 day storage, the values of antioxidant activity decreased in all the samples investigated, especially in the samples containing rose hip powder. Most probably, the carotenoid content decreased due to geometric isomerization and oxidation during the storage of the gingerbreads [41]. Despite this decrease, at every moment, the AA was higher in the samples with rose hip compared to the control, and was more significant with the 4% than with the 2% RHP addition (Table 4).

The sensory testing of the gingerbreads, performed by the specialized tasting panel, was necessary in order to predict the acceptance level of the product by consumers. By comparing the samples containing rose hip with the control, it can be noted that RHP has a general positive influence on all sensorial parameters, especially on taste, odor and color (which are improved for both RHP concentrations at every tested moment). There is a more important increase in the total sensory score of the 2% RHP biscuits (24.62) than the 4% RHP ones (23.81), compared to control (23.18). Therefore, rose hip powder has a positive influence on the organoleptic properties of the gingerbreads, especially when used in lower concentrations (2%). The gingerbreads containing 4% rose hip powder presented a strong specific smell and taste of rose hips, which makes the total sensory score of 4% RHP less important than in the case of 2% RHP. The consistency of the control changed in the first 25 days of storage; the biscuits lost their freshness and became dry. In the case of 2% RHP and 4% RHP, the modification of the organoleptic indices was non-essential, and the total sensory scores were 23.4 and 22.8, respectively. Over the next 20 days, the organoleptic indices changed and, as a result, the investigated samples summed 20.42 (control), 21.8 (2% RHP) and 21.0 (4% RHP). Thus, the concentration of rose hip powder of 2% is optimal, while the optimal shelf life for first-rate organoleptic scores is 25 days.

**Figure 5:**
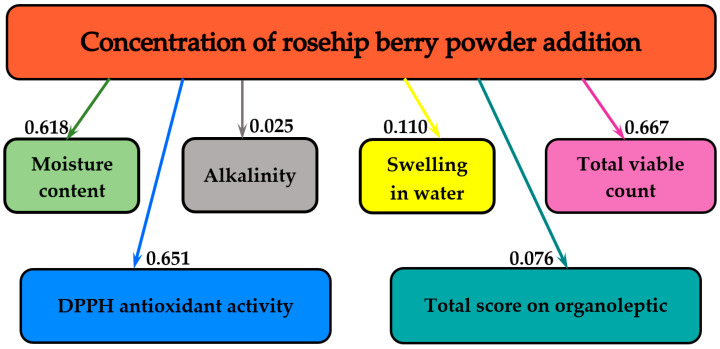
The influences of various quantities of rose hip powder on gingerbread physicochemical and organoleptic parameters, DPPH antioxidant activity and total viable count.

Figure 5 summarizes the influences of the different amounts of added rose hip powder on the physicochemical indicators (moisture content, alkalinity, swelling index), the total score of organoleptic indices, the *in vitro* DPPH antioxidant activity, and the microbiological stability (TVC) of the biscuits. The values of mutual influence are presented on the arrows.

As shown in Figure 5, the greatest influence of the concentration of rose hip powder was observed on total viable count and antioxidant activity, with the mutual information of 0.667 and 0.651 bits, respectively. On a descending scale, the next most significant influence was on the moisture content (0.618 bits). The smallest influence concerns the alkalinity (0.025 bits).

**Figure 6:**
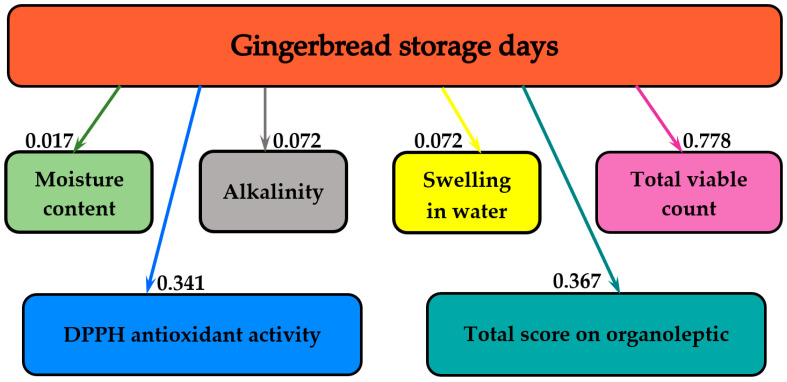
The informational analysis for the influence of storage days on gingerbread physicochemical and organoleptic parameters, DPPH antioxidant activity and total viable count.

Figure 6 summarizes the influence of storage on the gingerbread’s physicochemical indicators (moisture content, alkalinity, swelling in water), the total score of organoleptic indices, the *in vitro* DPPH antioxidant activity and the microbiological stability (TVC).

Figure 6 shows that the greatest influence of the storage duration was observed for total viable count, with the mutual information value of 0.778 bits. The antioxidant activity and total organoleptic score were influenced by 0.341 and 0.367 bits, respectively. Alkalinity and swelling in water were both influenced by 0.072. Storage time had the smallest influence on the moisture content, with the mutual information of 0.017 bits.

## 3. Materials and Methods

### 3.1. Chemical Materials

The ABTS reagent was provided by Alfa Aesar, the Folin–Ciocalteu reagent was purchased from Merck (Darmstadt, Germany), and (+)-catechin 98%, quercetin, caffeic acid, syringic acid, ferulic acid, gallic acid (98%), protocatechuic acid, gentisic acid, parahydroxybenzoic acid, salicylic acid (99.9%), *para*-coumaric acid, *trans*-5-*O*-caffeoylquinic) acid, epicatechin, vanillic acid, *cis*-resveratrol, ferulic acid methyl ester and DPPH were obtained from Sigma- Aldrich (Darmstadt, Germany, Tokyo, Japan, Shanghai, China). methyl 4-hydroxy-3-methoxycinnamate (99%) and sinapic acid (98%) were purchased from Alfa Aesar (Kandel, Germany). Procyanidin B1, procyanidin B2, polydatin, hyperoside, carotenoid standards, β-carotene, lycopene, β-cryptoxanthin, lutein, and zeaxanthin were purchased from Extrasynthese (Genay, France). Trans-resveratrol was purchased from TCI Europe (Zwijndrecht, Belgium). The acetonitrile and the formic acid were purchased from Merck. Quercetin (>95%) was obtained from Sigma-Aldrich (Bangalore, India). All spectrophotometric measurements were performed on the Analytik Jena Specord 200 Plus (Jena, Germany) spectrophotometer.

### 3.2. Biological Material

Dog rose (*Rosa canina* L.) fruits of the variety “Visocovitaminnii” were harvested at the end of August, by the company “Rose Line” SRL (Țaul, Republic of Moldova), which has its own plantations over 100 ha in Țaul village in the Dondușeni district, located in the northern part of the Republic of Moldova (48°12′57″ latitude, 27°40′22″ longitude and altitude of 226 m above sea level). The seeds were separated from the pulp and used for oil preparation, while the remaining pulp was dried at a temperature of 65 ± 1 °C to a humidity of 7.1 ± 0.1%, crushed to powder with the median particle (d50) of 140 ± 15 µm, then sieved.

The titratable acidity, dry weight and soluble solids of the fresh hips were determined using the methods described in ISO 750:1998 [42], the Handbook of Food Analysis [43] and ISO 2173:2003 [44], respectively.

### 3.3. Extract Characterization

The extraction was performed in 50% ethanol (1:10 ratio) under stirring at 60 rpm for 30 min at room temperature. After filtration, the polyphenol composition and the antioxidant activity were determined. Experiments on the influence of salts and pH were performed to establish optimal food matrix conditions. Before its addition in sugar syrup for glazing in gingerbread, the extract was concentrated in a rotary evaporator at 65.0 ± 1.0 °C until the mass fraction of dry substance reached 73.1 ± 0.1%. The extract was stored in glass bottles at 4.0 ± 1.0 °C, in the dark.

#### 3.3.1. Antioxidant Activity by Reaction with ABTS Radical

The antioxidant activity of the extracts was measured using the method described by Re et al. (1999) [45]. The results were expressed as mmol trolox equivalent (TE)/L from a calibration curve (0–2000 μmol/L) with trolox.

#### 3.3.2. Antioxidant Activity by Reaction with DPPH Radical

The method described by Brand-Williams et al. [46] was also used to determine antioxidant activity. The results were expressed as mmol TE/100 g from a calibration curve (0–250 μmol/L) with trolox.

#### 3.3.3. Total Polyphenols by Folin–Ciocalteu

To determine the total polyphenol content, the slightly modified method described by Ribereau-Gayon et al. [47] was used. The results for total polyphenols were calculated from a calibration curve of gallic acid (0–500 mg/L) and expressed in equivalents of gallic acid (GAE). The total content of flavonoids was also determined using precipitation with formaldehyde [48]. The results were expressed as quercetin equivalents (QE) based on a calibration curve (0–500 mg/L) of quercetin.

#### 3.3.4. Total Polyphenols by Abs 280

The total polyphenol content was also determined by measuring the absorbance at 280 nm. The result was expressed as equivalent of gallic acid (GAE) by construction of a calibration curve (0–50 mg/L), following the method described by Ribereau-Gayon et al. [47].

#### 3.3.5. Total Cinnamic Acids

The content of total cinnamic acids was determined by reading the absorbance at 320 nm after the acidification and the dilution of the sample with ethanol and HCl (2%). The results were expressed as caffeic acid equivalents (CAE) based on a calibration curve (0–50 mg/L) of caffeic acid [24,48].

#### 3.3.6. Total Flavonols

The content of total flavonols was determined by reading the absorbance at 360 nm after the acidification and the dilution of the sample with acidified ethanol and HCl (2%). The results were expressed as quercetin equivalents (QE) based on a calibration curve (0–50 mg/L) of quercetin [24,49].

#### 3.3.7. HPLC Analysis of Polyphenols

The method described by Cristea et al. [21] was used to determine the content of individual polyphenols. Agilent 1100 Series HPLC was employed and the gradient was optimized using trifluoroacetic acid (TFA) as an eluent with acidification of 1% CH_3_OH (A channel) and 50% CH_3_OH (B channel) acidified to 2.15 pH with TFA. The column system was composed of a pre-column SecurityGuard ULTRA Cartridges HPLC C18 for 4.6 mm ID coupled to Kinetex 5 μm C18 100 Å 250 × 4.6 mm columns manufactured by Phenomenex at 35 °C. The injection volume was 20 μL and the run time 90 min. The phases were A—H_2_O:CH_3_OH (99:1) and B—H_2_O:CH_3_OH (50:50), with a flow of 1.5 mL/min and detection was carried out at 256, 280, 324, and 365 nm. The gradient of elution was as follows: 100% (A) for 10 min; 82% (A) and 18% (B) for the next 10 min; 70% (A) and 30% (B) for 10 min; 65% (A) and 35% (B) for 6 min; 40% (A) and 60% (B) for 15 min; 20% (A) and 80% (B) for 5 min; 100% (B) for 15 min and 100% (A) for 10 min. The content of specific polyphenols was determined by comparison of retention times and peaks with the ones from the chromatogram of a synthetic mix containing the standards listed in Table 5.

#### 3.3.8. Carotenoid Extraction

The carotenoid extract was obtained by the method described by Ghendov-Mosanu et al. [23]. Subsequently, the obtained extracts were saponified with 30% methanolic KOH in the dark. To remove soaps and alkalis, the solution was washed several times with saturated sodium chloride solution and distilled water. The carotenoid-containing supernatant was dried and evaporated to dryness using a rotary evaporator. The samples were kept at −20 °C until use. Samples for analysis were dissolved in ethyl acetate and filtered through 0.45 mm PTFE (polytetrafluoroethylene) filters. Subsequently, the samples were analyzed by RP-HPLC [50]. All extractions were done in triplicate.

#### 3.3.9. Separation of Carotenoids by RP-HPLC

RP-HPLC analysis of carotenoids was performed on a Shimadzu LC-20AT with an SPD-M20A diode array detector (DAD) (Shimadzu Corporation, Kyoto, Japan). An YMC C30 column (250 × 4.6 mm; 5 μm) was used and the mobile phases consisted of the following: solvent A—methanol/tert-butyl methyl ether/water (83:15:2) and solvent B—methanol/tert-butyl methyl ether/water (8:90:2). The elution gradient was as follows: 0 min 0% solvent B, 20 min 0% B; 130 min –82% B; 132 min 0% B, followed by equilibration of column for 10 min. The flow rate was fixed at 0.8mL/min and the DAD detector was set at 450 nm. The identification of carotenoids from rose hip samples was carried out by the comparison of the UV–VIS spectra and the retention times of the sample peaks with those of the standard solutions, Table 6.

#### 3.3.10. Color Parameters (CIELab)

The CIELab parameters as defined by International Commission on Illumination/Commission Internationale de l’Eclairage were determined using the Specord 200 Plus (Jena, Germany) spectrophotometer. The transmittance of all the samples was measured between 380 nm and 780 nm, every nm, in an optical glass cuvette with the path length of 1 mm, using distilled water as reference, D65 as illuminant and the observer at 10° [51].

### 3.4. Study of Factors Influencing the Extract Properties

#### 3.4.1. Effects of Salts

NaCl, CaCl_2_ and KNO_3_ were added in different concentrations, i.e., 0.001 M, 0.01 M, and 0.1 M. The extracts were then stored at 4 °C for 12 h, after which the antioxidant activity and the color parameters (CIELab) were measured.

#### 3.4.2. Effects of pH Variations

The pH of the extract was adjusted to the values 2.5; 3.8; 5.4; 7.3, and 8.7, then stored at 4 °C for 12 h. The antioxidant activity and the color parameters (CIELab) were measured for each pH.

### 3.5. Gingerbread Making

Samples of gingerbread were made using 2% and 4% rose hip powder (2% RHP and 4% RHP) to determine its effects on the organoleptic, physicochemical, and microbiological indicators of the gingerbread. The rose hip powder was added directly to the wheat flour. The control sample was prepared without adding any rose hip material. Premium quality wheat flour, sugar, honey, eggs, ammonium bicarbonate, and pure water were used in gingerbread making. The sugar was dissolved in hot water (93 ± 2 °C) while mixing, then boiled for 30 min at 105 ± 1 °C, and cooled to 33 ± 1 °C. The eggs mix and honey were added to the cooled syrup and mixed. Afterwards, flour with/without rose hip powder and bicarbonate was poured in slowly while stirring. The dough, kneaded for 15–20 min, was left to rest for 2 h at 20 °C. The dough, shaped into gingerbread biscuits, with a thickness of 20 mm, was baked in the oven at 180 ± 1 °C for 13 ± 1 min. The baked gingerbreads were cooled and glazed with sugar syrup. To prepare the syrup, sugar and water in a ratio of 1:0.4 were mixed and boiled for 30 min so that at the end of boiling the syrup had a temperature of 113 ± 1 °C. The boiled syrup was cooled to 55–60 °C, after which the concentrated extract of rose hip in a quantity of 2% was added to the syrup mass. After glazing, the biscuits were dried at 63 ± 1 °C for 7 ± 1 min, and packed and stored in a dry place at room temperature. The specific quantities of the manufacturing recipe were 100.0 g wheat flour, 50.0 g sugar, 25 g honey, 12 g chicken eggs mix, 1.7 g ammonium bicarbonate and water (11.1 mL (control), 11.4 mL (2% RHP), 11.7 mL (4% RHP)).

### 3.6. Gingerbread Analysis

The gingerbread was analyzed on the 1st, 25th and 45th days from production date in order to study the parameters’ evolution during storage.

#### 3.6.1. Physicochemical Analysis

The moisture was assessed by using the weight loss method. The sample was heated at 105 °C to a constant weight [52]. Titration with hydrochloric acid 0.1 N using bromothymol blue as indicator was used to determine alkalinity [53]. The swelling in water of the gingerbread was determined by immersion in water [54]. This method is based on the measurement of the mass increase of flour confectionery when immersed in water at a temperature of 20 °C for 2 min.

#### 3.6.2. Microbiological Analysis

Standard ISO 4833-2:2013/COR 1:2014 [36] was followed when performing the microbiological analysis. The horizontal method for the enumeration of microorganisms which form colonies after plating and incubation was employed. The total viable count (TVC) of mesophilic aerobic organotrophic bacteria was determined following incubation at 30 °C for 4–72 h using nutrient agar.

#### 3.6.3. Sensory Analysis

Standard ISO 6658:2017 [55] was followed when performing the sensory analysis of the products. Appearance, color, odor, taste, and consistency were assessed using the 5-point system by an expert panel of eleven trained food technologists. The 5-point assessment system includes the following scores: 5—very good; 4—good; 3—satisfactory; 2—poor; 1—bad and 0—very bad.

#### 3.6.4. *In Vitro* Antioxidant Activity of Gingerbread

The DPPH radical scavenging assay was used to measure the antioxidant activity *in vitro*, in the conditions of gastric digestion. Pepsin (150 mg/100 g of product) was used to simulate gastric digestion at pH = 2.0 ± 0.1 (1.5 M HCl) and 37.0 ± 0.1 °C, under stirring for 2 h at 600 rpm. Afterwards, the mixture was centrifuged at 6000 rpm for 10 min and filtered. The DPPH radical scavenging activity of the clear solution was measured at room temperature (20 ± 1 °C) following the method of Brand-Williams et al. [46]. The results were expressed as µmol TE/100 g from a calibration curve (0–250 μmol/L) with trolox.

### 3.7. Statistical Analysis

The mean values and the standard deviations were calculated from 3 parallel experiments. One-way and two-way ANOVA, and post-hoc Tukey tests, were used to distinguish between means and evaluate the results. The considered significance level was *p* ≤ 0.05. The statistical calculations were performed using IBM SPSS Statistics 23, and MathWorks (Natick, MA, USA). The used method which facilitates the evaluation of the influences among various measured parameters was MATLAB. Entropy and information were the main concepts employed, while the bit was the unit of measure [56,57].

## 4. Conclusions

Rose hips of the variety “Visocovitaminnii” are characterized by a high content of polyphenols, i.e., 5484 mg GAE/100 g, of which 1199 mg GAE/100 g are flavonoids, and a high antioxidant activity. The main phenolic compounds found in this study were procyanidin B1, chlorogenic acid (*trans*-5-*O*-caffeoylquinic acid), epicatechin, procyanidin B2, gallic acid, salicylic acid, catechin, etc. The total carotenoid content was 64.03 mg/100 g dry weight. The carotenoid complex includes all-*trans*-β-carotene, all-*trans*-lycopene, zeaxanthin; α-cryptoxanthin, β-cryptoxanthin, rubixanthin, *cis*-β-carotene, *cis*-γ-carotene, and *cis*-lycopene. The presence of CaCl_2_ and NaCl lowered the value of antioxidant activity while KNO_3_ did not produce any changes. Moreover, all the salts produced significant modifications of the red/green parameter and shifted the color towards greener tones. They also increased the yellowness. Lowering the pH to 2.5 decreased the antioxidant activity by around 10 mmol TE/L, while the L* value was lowered from around 96 to around 80 at pH = 5.4. Moreover, at pH = 3.8, the redness of the extract was enhanced.

RHP could be used as a natural food additive, especially in organic products. Beneficial effects of the addition of RHP on the properties of gingerbread have been observed. RHP of 2% and 4% improved the physicochemical and sensory characteristics of gingerbread, and ensured antioxidant activity and microbiological stability by reducing total viable count.

Given that the analyzed RHP came from the pulp of *Rosa canina* L., obtained by separating the seeds to produce oil, the present study raises the current level of knowledge on the efficient use of plant raw materials by increasing sustainability.

## Figures and Tables

**Table 1:** The content of total and individual polyphenols, carotenoids and the antioxidant activity of the rose hip dry powder used for experiments (the results are expressed as means ± standard deviations of three experiments).

Indices	Quantity
Total polyphenols (Folin–Ciocalteu), mg gallic acid equivalents (GAE)/100 g	5484 ± 1001
Total polyphenols (Abs280), mg GAE/100 g	2968 ± 21
Total flavonoids, mg quercetin equivalents (QE)/100 g	2130 ± 39
Cinnamic acids, mg caffeic acid equivalents (CAE)/100 g	224 ± 12
Flavonols, mg QE/100 g	194 ± 7
Procyanidin B1, mg/100 g	29.1 ± 1.7
Chlorogenic (*trans*-5-*O*-caffeoylquinic) acid, mg/100 g	10.5 ± 0.2
Epicatechin, mg/100 g	5.7 ± 1.2
Procyanidin B2, mg/100 g	5.2 ± 1.1
Gallic acid, mg/100 g	5.1 ± 0.02
Salicylic acid, mg/100 g	5.0 ± 0.0
Catechin, mg/100 g	4.6 ± 0.4
Ferulic acid, mg/100 g	3.3 ± 1.3
Gentisic acid, mg/100 g	2.9 ± 0.4
Protocatechuic acid, mg/100 g	2.1 ± 0.2
*p*-Hydroxybenzoic acid, mg/100 g	2.1 ± 0.8
Polydatin, mg/100 g	1.6 ± 1.2
Sinapic acid, mg/100 g	0.3 ± 0.1
*p*-Coumaric acid, mg/100 g	0.2 ± 0.1
Vanillic acid, mg/100 g	0.2 ± 0.1
*Cis*-resveratrol, mg/100 g	0.1 ± 0.0
*m*-Hydroxybenzoic acid, mg/100 g	Traces
Quercetin, mg/100 g	Traces
Caffeic acid, mg/100 g	Traces
*Trans*-resveratrol, mg/100 g	nd
Hyperoside, mg/100 g	nd
Ferulic acid methyl ester, mg/100 g	nd
Syringic acid, mg/100 g	nd
Total carotenoids, mg/100 g	64.03 ± 1.45
Zeaxanthin, mg/100 g	1.99 ± 0.14
α-Cryptoxanthin, mg/100 g	1.28 ± 0.08
β-Cryptoxanthin, mg/100 g	0.74 ± 0.03
Rubixanthin, mg/100 g	1.22 ± 0.24
*cis*-β-Carotene, mg/100 g	0.45 ± 0.05
all-*trans*-β-Carotene, mg/100 g	1.84 ± 0.27
*cis*-γ-Carotene, mg/100 g	0.46 ± 0.02
*cis*-Lycopene, mg/100 g	0.38 ± 0.01
all-*trans*-Lycopene, mg/100 g	2.72 ± 0.18
ABTS Antioxidant activity, mmol trolox equivalents (TE)/100 g	41.54 ± 0.33
DPPH Antioxidant activity, mmol TE/100 g	140.8 ± 1.4

nd = not detected, ABTS = 2,2′-azino-bis-3-ethylbenzthiazoline-6-sulphonic acid, DPPH = 2,2-diphenyl-1-picryl-hydrazyl-hydrate.

**Table 2:** Change of CIELab color parameters for different salts and different concentrations (standard deviations are based on three replicates).

Salt and Concentration	L*	A*	B*	C*	H*, °	ΔE*
Control	92.42 ± 0.03 ^a^	0.51 ± 0.01 ^d^	18.30 ± 0.07 ^d^	18.30 ± 0.07 ^a^	88.42 ± 0.01 ^a,e^	-
NaCl, 0.001 M	95.63 ± 0.15 ^c^	−0.25 ± 0.02 ^b^	14.78 ± 0.26 ^a,b^	14.78 ± 0.26 ^b^	90.97 ± 0.18 ^a^	4.82 ± 0.22 ^a^
NaCl, 0.01 M	95.95 ± 0.24 ^c^	−0.30 ± 0.03 ^a,b^	14.86 ± 0.30 ^b^	14.87 ± 0.30 ^b^	91.09 ± 0.16 ^a,c^	18.36 ± 0.31 ^b^
NaCl, 0.1 M	96.23 ± 0.10 ^c^	−0.35 ± 0.02 ^a^	14.62 ± 0.12 ^a,b^	14.63 ± 0.12 ^b^	91.36 ± 0.12 ^c^	5.39 ± 0.09 ^c,e^
KNO_3_, 0.001 M	95.97 ± 0.20 ^c^	−0.04 ± 0.01 ^c^	15.47 ± 0.03 ^c^	15.47 ± 0.03 ^b^	90.14 ± 0.03 ^b^	5.37 ± 0.17 ^c,e^
KNO_3_, 0.01 M	95.04 ± 0.13 ^c^	−0.03 ± 0.03 ^c^	15.44 ± 0.07 ^c^	15.44 ± 0.07 ^b^	90.07 ± 0.10 ^b,d^	4.57 ± 0.10 ^a^
KNO_3_, 0.1 M	94.42 ± 0.07 ^b^	0.06 ± 0.02 ^c^	15.91 ± 0.09 ^c^	15.91 ± 0.09 ^b^	89.77 ± 0.07 ^d^	3.15 ± 0.04 ^d^
CaCl_2_, 0.001 M	95.97 ± 0.20 ^c^	−0.29 ± 0.03 ^a,b^	14.32 ± 0.06 ^a^	14.33 ± 0.06 ^b^	91.18 ± 0.13 ^a,c^	5.03 ± 0.17 ^a,e^
CaCl_2_, 0.01 M	95.70 ± 0.34 ^c^	−0.26 ± 0.01 ^b^	14.80 ± 0.19 ^a,b^	14.80 ± 0.19 ^b^	90.99 ± 0.16 ^a^	4.86 ± 0.33 ^a,e^
CaCl_2_, 0.1 M	96.02 ± 0.08 ^c^	−0.26 ± 0.01 ^b^	14.82 ± 0.22 ^a,b^	14.82 ± 0.22 ^b^	91.01 ± 0.05 ^a,c^	5.07 ± 0.16 ^a,c,e^

Different letters ^(a–e)^ designate statistically different results (*p* ≤ 0.05). L*—luminosity; a*—red/green component; b*—yellow/blue component; C*—chromaticity; H*—hue angle; ΔE*—overall difference of color.

**Table 3:** CIELab color parameters’ dependence on pH (results are expressed as means ± standard deviation).

CIELab Parameters	L*	a*	b*	C*	H*, °	ΔE*
Control for 2.5	97.3 ± 0.0 ^a^	−0.1 ± 0.0 ^a^	6.0 ± 0.0 ^a^	6.0 ± 0.0 ^a^	91.27 ± 0.05 ^a^	0.85 ± 0.14 ^a^
pH = 2.5	98.1 ± 0.1 ^a^	−0.3 ± 0.0 ^a^	6.2 ± 0.1 ^a^	6.3 ± 0.1 ^a^	92.55 ± 0.20 ^b^	
Control for 3.8	96.0 ± 0.1 ^a^	−0.1 ± 0.0 ^a^	6.8 ± 0.0 ^a^	6.8 ± 0.0 ^a^	90.88 ± 0.14 ^a^	2.29 ± 3.53 ^a^
pH = 3.8	94.0 ± 3.5 ^a^	0.1 ± 0.3 ^a^	7.9 ± 0.9 ^a^	7.9 ± 0.9 ^a^	149.32 ± 0.18 ^a^	
Control for 5.4	95.9 ± 0.2 ^a^	−0.2 ± 0.1 ^a^	9.1 ± 0.0 ^a^	9.1 ± 0.0 ^a^	88.42 ± 0.01 ^a^	16.30 ± 0.24 ^b^
pH = 5.4	79.6 ± 0.1 ^b^	0.1 ± 0.0 ^a^	9.2 ± 0.2 ^a^	9.2 ± 0.2 ^a^	89.54 ± 0.28 ^b^	
Control for 7.3	95.9 ± 0.2 ^a^	−0.2 ± 0.1 ^a^	9.1 ± 0.0 ^a^	9.1 ± 0.0 ^a^	91.06 ± 0.22 ^a^	1.78 ± 0.33 ^a^
pH = 7.3	95.7 ± 0.5 ^a^	1.1 ± 0.0 ^a^	10.3 ± 0.1 ^b^	10.3 ± 0.1 ^a^	83.65 ± 0.05 ^b^	
Control for 8.7	96.9 ± 0.1 ^a^	−0.12 ± 0.1 ^a^	7.3 ± 0.1 ^a^	7.3 ± 0.1 ^a^	91.06 ± 0.22 ^a^	8.46 ± 1.21 ^c^
pH = 8.7	92.7 ± 1.2 ^b^	2.4 ± 0.2 ^b^	14.2 ± 0.6 ^b^	14.4 ± 0.6 ^a^	80.27 ± 0.45 ^b^	

Different letters ^(a–c)^ designate significantly different results between pairs of test and control for each value of pH (*p* ≤ 0.05). L*—luminosity; a*—red/green component; b*—yellow/blue component; C*—chromaticity; H*—hue angle; ΔE*—overall difference of color.

**Table 4:** Physicochemical and microbiological quality indicators, sensory profile, and antioxidant activity of gingerbread with added rose hip powder compared to the control (the results are presented as means ± standard deviation).

Quality Indicators	Gingerbread								
	Control			2% RHP			4% RHP		
	1st Day	25th Day	45th Day	1st Day	25th Day	45th Day	1st Day	25th Day	45th Day
Moisture content, %	10.31 ± 0.12 ^a^	10.0 ± 0.14 ^a^	9.45 ± 0.08 ^b^	14.01 ± 0.15 ^c^	13.41 ± 0.17 ^d^	12.96 ± 0.14 ^e^	14.68 ± 0.18 ^f^	13.87 ± 0.15 ^c,d^	13.42 ± 0.16 ^g^
Alkalinity, degrees	1.99 ± 0.01 ^a^	1.94 ± 0.01 ^b^	1.90 ± 0.01 ^c^	1.97 ± 0.01 ^a^	1.93 ± 0.01 ^b^	1.89 ± 0.01 ^c^	1.94 ± 0.01 ^b^	1.89 ± 0.01 ^c^	1.85 ± 0.01 ^d^
Swelling in water, %	153 ± 6 ^a^	158 ± 7 ^a^	160 ± 10 ^a^	157 ± 6 ^a^	160 ± 8 ^a^	165 ± 5 ^a^	161 ± 9 ^a^	169 ± 14 ^a^	174 ± 11 ^a^
Total viable count (TVC), % of the maximum admissible number *	9.0 ± 1.3 ^a^	15.4 ± 1.9 ^b^	26.3 ± 2.7 ^c^	5.3 ± 0.6 ^a^	14.9 ± 1.5 ^b^	25.4 ± 2.1 ^c^	4.2 ± 0.5 ^a^	13.0 ± 1.6 ^b^	21.7 ± 2.1 ^c^
DPPH^•^ antioxidant activity, µM TE/100 g	*n/a*	*n/a*	*n/a*	12.07 ± 1.94 ^a^	7.74 ± 2.11 ^b,e^	3.21 ± 0.96 ^c^	16.58 ± 1.15 ^d^	11.74 ± 1.91 ^a,b^	6.56 ± 0.69 ^c,e^
Sensory profile total score	23.18 ± 0.17 ^a^	21.53 ± 0.20 ^b,f^	20.42 ± 0.09 ^c^	24.62 ± 0.20 ^d,f^	23.40 ± 0.10 ^a,e^	21.80 ± 0.10 ^b^	23.81 ± 0.17 ^e^	22.80 ± 0.10 ^a^	21.00 ± 0.40 ^f^
Appearance	4.35 ± 0.05 ^a,f^	4.21 ± 0.02 ^a,b,g^	4.10 ± 0.02 ^b,g^	4.79 ± 0.04 ^c^	4.60 ± 0.10 ^c,d^	4.30 ± 0.10 ^a,b,g^	4.66 ± 0.07 ^c,e^	4.50 ± 0.10 ^c,f^	4.10 ± 0.10 ^g^
Taste	4.69 ± 0.04 ^a,d,e^	4.48 ± 0.02 ^d^	4.13 ± 0.02 ^b,c^	4.97 ± 0.06 ^e^	4.80 ± 0.10 ^a,e^	4.50 ± 0.10 ^d^	4.80 ± 0.20 ^a,e^	4.60 ± 0.10 ^a,d^	4.20 ± 0.10 ^c^
Odor	4.74 ± 0.05 ^a,f,g^	4.22 ± 0.01 ^b,c,d,e^	4.05 ± 0.02 ^c,e^	5.00 ± 0.00 ^a^	4.60 ± 0.10 ^f,g^	4.40 ± 0.10 ^d,f,g,e^	4.95 ± 0.05 ^a^	4.50 ± 0.10 ^g^	4.30 ± 0.20 ^e,g^
Color	4.57 ± 0.06 ^a,d^	4.32 ± 0.01 ^a,b^	4.14 ± 0.02 ^b^	4.97 ± 0.06 ^c,d^	4.80 ± 0.10 ^d^	4.40 ± 0.10 ^a,b^	4.63 ± 0.12 ^a,d^	4.60 ± 0.10 ^a,d^	4.37 ± 0.25 ^a,b^
Consistency	4.83 ± 0.06 ^a,f^	4.30 ± 0.20 ^b,c,d,e^	4.00 ± 0.10 ^c,d^	4.90 ± 0.10 ^a^	4.60 ± 0.10 ^a,b,f^	4.20 ± 0.10 ^d,f,e^	4.77 ± 0.06 ^f,e^	4.60 ± 0.10 ^b,f,e^	4.03 ± 0.15 ^e^

Different letters (^a–g^) designate statistically different results (*p* ≤ 0.05); *n/a* = no activity; * = nutrient agar.

**Table 5:** Polyphenols used as standards in HPLC analysis and their retention times.

Compound	Max Absorption (nm)	Retention Time (min)
Gallic acid	280	5.294
Protocatechuic acid	256	9.267
*p*-hydroxybenzoic acid	256	13.918
Gentisic acid	324	15.531
Procyanidin B1	280	16.704
*m*-hydroxybenzoic acid	280	17.989
Catechin	280	18.53
Vanillic acid	256	20.319
Caffeic acid	324	20.485
*trans*-5-*O*-caffeoylquinic acid	324	22.871
Procyanidin B2	280	23.433
Syringic acid	280	25.002
Epicatechin	280	26.836
*p*-coumaric acid	324	29.695
Ferulic acid	324	36.233
Salycilic acid	280	36.995
Polydatin	280	38.234
Sinapic acid	324	38.564
Hyperoside	280	47.305
*trans*-resveratrol	324	49.333
*cis*-resveratrol	324	57.089
Ferulic acid methyl ester	365	57.754
Quercetin	256	65.278

**Table 6:** Carotenoids used as standards in RP-HPLC analysis and their retention times.

Compound	Max Absorption (nm)	Retention Time (min)
Zeaxanthin	426, 450, 476	10.015
α-Cryptoxanthin	420, 445, 473	33.249
β-Cryptoxanthin	428, 451, 476	35.924
Rubixanthin	427, 460, 490	44.551
*cis*-β-Carotene	424, 446, 472	69.650
all-*trans*-β-Carotene	421, 452, 478	74.212
*cis*-γ-Carotene	428, 456, 477	77.989
*cis*-Lycopene	444, 467, 496	84.343
all-*trans*-Lycopene	448, 471, 503	94.026
